# A Schema Therapy–Based eHealth Program for Patients with Borderline Personality Disorder (priovi): Naturalistic Single-Arm Observational Study

**DOI:** 10.2196/10983

**Published:** 2018-12-17

**Authors:** Gitta Anne Jacob, Andrea Hauer, Sandra Köhne, Nele Assmann, Anja Schaich, Ulrich Schweiger, Eva Fassbinder

**Affiliations:** 1 GAIA AG Hamburg Germany; 2 Department of Psychiatry and Psychotherapy University of Lübeck Lübeck Germany

**Keywords:** borderline personality disorder, eHealth, mHealth, psychotherapy, schema therapy

## Abstract

**Background:**

Electronic health (eHealth) programs have been found to be effective in treating many psychological conditions. However, regarding borderline personality disorder (BPD), only a few eHealth programs have been tested, involving small interventions based on the dialectical behavior therapy treatment approach. We investigated priovi, a program based on the schema therapy (ST) approach. priovi is considerably more comprehensive than prior programs, offering broad psychoeducation content and many therapeutic exercises.

**Objective:**

We tested the acceptability and feasibility of priovi in 14 patients with BPD as an add-on to individual face-to-face ST.

**Methods:**

Patients received weekly individual ST and used priovi over a period of 12 months. We assessed BPD symptom severity using self-reported and interview-based measures. Qualitative interviews were conducted with both patients and therapists to assess their experiences with priovi.

**Results:**

BPD symptoms improved significantly (Cohen *d*=1.0). Overall, qualitative data showed that priovi was positively received by both patients and therapists. Some exercises provoked mild anxiety; however, no serious threat to safety was detected.

**Conclusions:**

priovi is a potentially helpful and safe tool that could support individual ST. It needs to be further tested in a randomized controlled study.

**Trial Registration:**

German Clinical Trials Register DRKS00011538; https://www.drks.de/drks_web/navigate.do? navigationId=trial.HTML&TRIAL_ID=DRKS00011538 (Archived by WebCite at http://www.webcitation.org/74jb0AgV8)

## Introduction

Electronic health (eHealth) applications have been found to be helpful in treating many psychological conditions. They have been extensively studied for common disorders (eg, depression or anxiety disorders) [1,2] and a broad spectrum of less frequent conditions, such as eating disorders [3,4] or posttraumatic stress disorder [5]. However, only a few studies have so far investigated eHealth applications in relation to people with borderline personality disorder (BPD).

BPD is a severe, often chronic psychological condition. Affective instability, self-injuring behavior, impulsivity, and identity problems are the hallmark symptoms of BPD. Those affected suffer severely, use a lot of psychological and psychiatric treatment, and are often severely impaired in their social and professional functioning. BPD also constitutes a high economic burden on society [6,7], with disease prevalence in the general population estimated to range from 1.2% to 2% [8,9].

Several psychological treatments for BPD have been developed and positively tested, including dialectical behavior therapy (DBT), schema therapy (ST), mentalization-based therapy (MBT), and transference-focused psychotherapy [10]. These treatments notably take a long time to administer, requiring several years for completion. Meanwhile, implementation and dissemination are slow, and most patients with BPD do not receive these treatments [11]. eHealth applications may offer innovative, cost-effective ways to provide evidence-based treatment for more patients with BPD [12].

All existing digital interventions for BPD are related to teaching DBT skills. The DBT Coach mobile phone app offers coaching for skills use and has been studied in 2 small uncontrolled pilot studies [13,14]. Results showed good feasibility, acceptability, and subjective patient evaluations of the DBT Coach as part of a standard DBT program. The EMOTEO (emotion–meteo [weather forecast]) mobile phone app has been tested for usability and efficiency with regard to the reduction of inner tension over 6 months in 16 patients with BPD. It was found to be user-friendly and efficient in reducing aversive tension [15]. Pocket Skills is another mobile phone app that teaches DBT skills. It was studied over a 4-week period in 73 individuals with mixed diagnoses (mainly depression, anxiety, and BPD) and enrolled in psychotherapy. It helped participants to engage in their DBT. They reported decreased depression and anxiety and increased DBT skill use [16]. Meanwhile, an internet-delivered DBT skills training intervention has been investigated over a 4-month period in a randomized controlled trial (RCT) with suicidal individuals who engaged in heavy episodic drinking. Compared with the waitlist, individuals in the internet-delivered DBT skills training intervention condition showed faster reductions in alcohol consumption [17]. Another pilot study examined the feasibility of 4 sessions of avatar therapy in virtual reality as an add-on to MBT in BPD [18]. Qualitative data suggest that avatar-MBT is acceptable to patients with BPD and has a positive impact. For ST, no Web-based intervention has been tested yet.

Thus, we developed priovi, which is based on ST. In ST, problematic BPD behaviors and symptoms are linked with “schema modes,” (ie, emotional states related to dysfunctional schemas such as mistrust or abuse or abandonment). The typical schema modes of patients with BPD are the vulnerable child mode (related to intense feelings of abandonment, sadness, anxiety, and mistrust), the angry or impulsive child mode (related to angry outbursts and impulsive behaviors), the punitive parent mode (related to self-devaluation and self-punishment), and the detached protector mode (related to problematic emotion-avoidance strategies such as dissociation, substance abuse, binge eating, and social withdrawal). The healthy adult mode (related to healthy functioning and relationships) is usually weak at the beginning of treatment. In the first part of treatment, patients learn to identify their schema modes and to understand their biographical background. Treatment goals in the following phase of therapy are also related to modes (ie, support and comfort the vulnerable child mode, help the angry child mode find better ways to deal with anger, control the punitive parent mode, and reassure the detached protector mode), so that patients can reduce their emotional avoidance and learn healthier ways to deal with emotions and relationships. To achieve these goals, mode-specific cognitive, experiential, and behavioral interventions are used. In addition, the therapy relationship, which is conceptualized as limited reparenting, is warm, caring, directive, and often non-Socratic. Several RCTs have shown the tremendous efficacy of ST in treating BPD [19]. It is currently recognized as one of the most promising approaches for treating severe disorders such as various personality disorders (PDs) [20] or forensic patients [21]. ST has shown low treatment dropout and high acceptance in both patients and therapists [19,22].

With priovi, we have developed the first ST-based Web-based intervention comprising broad psychoeducational content and several therapeutic exercises. If feasible, priovi could offer a lot of therapeutic content to patients with BPD and relieve therapists from discussing all the content in detail themselves, thereby saving therapy time and speeding up therapy substantially.

In this first pilot study on a Web-based ST intervention, we offered priovi to patients as an add-on to face-to-face ST. We investigated whether priovi is feasible and acceptable to patients and whether both patients and therapists found it helpful. The study was approved by the ethical committee of the University of Lübeck, Germany (AZ 14-038) and has been registered in the German Clinical Trials Register (DRKS-ID: DRKS00011538).

## Methods

### Subjects

We recruited patients from people asking for treatment in the outpatient center of the Department of Psychiatry and Psychotherapy at Lübeck University, Germany. The outpatient center treats chronic and severely ill patients. Textbox 1 show the inclusion and exclusion criteria. Regarding substance use, patients were included when they fulfilled only the criteria of substance abuse or were abstinent for >2 months.

There were 14 patients with BPD who began the trial. Of these, 1 patient was soon excluded, as she developed a psychotic episode shortly after the start of the trial, which was not related to ST treatment or priovi. Of the remaining 13 study participants, 9 completed all 4 assessments, 2 each missed assessments after 6 and 12 months, and 4 missed the assessment after 18 months.

Of the 13 study participants, 11 were females. The mean age was 28.4 (SD 8.3) years. There were 4 patients who had children, and 8 lived with a spouse. The average level of education was 10.8 years. Regarding the job situation, 4 were on long-term sick leave, 7 worked in a regular job, and 2 were unemployed. The patients had a high number of comorbid psychiatric conditions including on Axis I, a current major depressive disorder(11/13, 85%), posttraumatic stress disorder (10/13, 77%), social phobia (6/13, 46%), agoraphobia with panic disorder (5/13, 38%), generalized anxiety disorder (2/13, 15%), specific phobia (5/13, 38%), obsessive-compulsive disorder (3/13, 23%), bulimic disorder (6/13, 46%), and substance abuse (5/13, 38%). On Axis II, the comorbidities included obsessive-compulsive PD (5/13, 38%), avoidant PD (5/13, 38%), paranoid PD (3/13, 23%), and narcissistic PD (1/13, 8%). There were eleven patients (11/13, 85%) on psychopharmacological medication. All of them took antidepressants, 6 had an additional antipsychotic medication, and 1 patient used zopiclone.

With regard to prior treatments, only 2 patients were treatment-naïve, 11 patients had received prior medication (mean 8.5 [SD 5.9] different medications) and individual psychotherapy, and 9 had received group psychotherapy. Patients with prior treatments reported a mean of 5.5 inpatient treatments and 3.8 outpatient treatments. Note that not all these treatments necessarily focused on BPD, since many patients had also been treated for comorbid conditions, mainly depression and eating disorders.

### Intervention

All patients received weekly individual face-to-face sessions and priovi for 1 year. priovi is a dialogue-based program; all content is presented to the user in (written) therapeutic dialogue. The program is highly tailored to the individual user, with the therapeutic conversation evolving based on the user’s responses in the dialogue. Within the dialogue format, priovi communicates psychoeducational content, explains therapeutic techniques, and guides the user through exercises. Users can pause and continue sessions anytime they want. Apart from written text, priovi contains several audio guides and illustrations.

The first phase of the program covers psychoeducation on BPD symptoms, human needs, childhood abuse, and BPD-specific modes and emotions. All content is offered playfully, through the use of explanatory text, case examples, games, imagery exercises, comics, and illustrations. For example, after receiving psychoeducation about different modes, comic strips are presented showing interpersonal conflict situations. The user has to guess the modes involved in the reaction of the respective protagonists.

Phase II contains many mode-specific exercises tailored to the needs of the user. The order of exercises is fixed and follows the usual ST recommendations. However, the user can skip exercises when they do not feel prepared for them. For each mode, several (between 3 and 6) exercises are offered, with increasing difficulty. Users can repeat exercises or work on skipped exercises later on (Table 1).

Additional components of priovi include, to mention a few, an individual “mode-toolbox” with helpful strategies for each mode; a “glossary” with important terms and information; regular tracking of BPD symptoms, depression, and mood; and daily emails or text messages. Depending on the user’s approval, therapists can observe the progress of their patients in the “cockpit”—a clinician-facing interface that provides an overview of the patient’s sessions with priovi. In this feasibility study, therapists were instructed to monitor the “cockpit” before each session and support patients’ usage of priovi by discussing it on a regular basis.

**Inclusion and exclusion criteria.**

**Inclusion criteria**

- Primary borderline personality disorder diagnosis
- Borderline Personality Disorder Severity Index≥20
- Age≥18 years
- Fluency in German
- Willingness to participate
- Written informed consent

**Exclusion criteria**

- Current psychotic disorder
- Alcohol or benzodiazepine dependency requiring immediate detox treatment

**Table 1:** Example exercises in phase II of priovi.

Exercise type	Example
Exercises to overcome avoidant coping modes	Collecting individual pros and cons of these modes by selecting options from lists with typical pros and cons; imagery of nonavoidant behaviors with audio instructions
Exercises to soothe the vulnerable child mode	Collecting positive feedback from others and writing it into a personal diary; imagery exercises of caring for the inner child in a safe place, guided by audio instructions
Exercises to control the punitive parent mode	Collecting arguments against this mode by selecting options from a list with typical arguments; developing counter-arguments
Exercises to strengthen the healthy adult mode	Implementing healthy and pleasant behavior in real life, supported by audio instructions; dealing with conflicts and problems in real life, supported by audio instructions

priovi can be used on all Web-enabled screen devices. Once registered, access to the program lasts for 1 year. We recommend using it twice weekly for about half an hour. If the user complies with this recommendation, it takes them about 6 months to work through all dialogues. A more detailed description of priovi including some screenshots can be found elsewhere [12].

### Assessments

Assessments took place before the start of treatment and after 6, 12, and 18 months. We mainly report on the 1-year outcome, since priovi ended after 1 year, while individual treatment might have been continued.

### Borderline Personality Disorder Interview and Questionnaire

BPD severity was assessed with the Borderline Personality Disorder Severity Index (BPDSI)-IV, a well-established semistructured interview representing the severity and frequency of BPD manifestations over the last 3 months according to the Diagnostic and Statistical Manual of Mental Disorders, fifth edition [23-25]. In addition, we used the BPD Checklist short version, a 47-item self-report questionnaire assessing BPD symptom severity in the last month [26].

#### Qualitative Patient Interviews

In-depth qualitative semistructured guideline interviews were conducted to get more detailed insight into the patient’s experience with priovi. We interviewed 11 patients at the end of treatment. Interviews were conducted by 1 of the authors (SK) and 2 research assistants, digitally recorded and pseudonymized. A protocol paraphrasing the major information was typed out. There was no fixed time set for the interviews; the duration varied from 9 to 39 minutes.

#### Qualitative Therapist Interviews

In a similar way, all 6 study therapists (4 females, 2 males, mean age 34.7 [SD 3.7] years) were also interviewed. These interviews lasted on average 42 minutes (range 28-52 minutes).

### Analysis

We analyzed quantitative data using SPSS software with repeated measures analysis of variance with the last observation carried forward. Effect sizes were calculated using Cohen *d*. Correlations were calculated with Excel. Qualitative data were thematically analyzed, and interviews were interpreted by means of the qualitative content analysis sensu Mayring [27], extracting the core messages of the interviews.

## Results

### Principal Results

All patients used the program to a significant extent. The mean days of usage were 80.7 (SD 72, range 12-288), and total mean usage time was 19 (SD 10.9, range 6.2-40.3) hours. Patients tended to use the program over the entire year of treatment (range 68-365 days, mean 304 [SD 93] days).

### Borderline Personality Disorder Symptoms

Table 2 shows BPDSI and BPD-CL results over time. The last observation was carried forward in cases of missing data. BPDSI scores were reduced by 9.6 points over 1 year, equaling about Cohen *d*=1.0 (taking the mean [SD] of all assessments=9.7 as a reference). The BPD-CL was reduced by 29.9 (SD 25.6) points, equaling Cohen *d*=1.2. All changes were significant over time. Symptom changes were not correlated with usage time (*r*=.067).

### Qualitative Results

In the interviews, patients related to 6 positive and 5 negative categories regarding their experience with priovi (Table 3).

Therapists reported helpful functions of priovi and mentioned both positive and negative effects on the therapist, the therapy process, and the patients’ progress (Table 4).

**Table 2:** Borderline Personality Disorder Severity Index and Borderline Personality Disorder Checklist results.

Checklist	Baseline	6 months	12 months	18 months	*F*_3,36_^a^	*P* value^a^
BPDSI^b^, mean (SD)	33.3 (7.4)	26.6 (9.5)	23.6 (11.3)	22.3 (11.9)	6.62	.001
BPD-CL^c^, mean (SD)	129.0 (21.8)	106.7 (26.7)	98.8 (31.1)	95.1 (28.4)	7.21	.001

^a^*F* and *P* values of repeated measurements analysis of variance.

^b^BPDSI: Borderline Personality Disorder Severity Index.

^c^BPD-CL: Borderline Personality Disorder Checklist.

**Table 3:** Positive and negative categories reported by 11 patients with regard to their experiences with priovi.

Category type	Category	n (%)^a^	Patient experience
Positive	Local and temporal flexibility	6 (55)	The constant availability of priovi made patients feel safe, helped them to optimize their learning process, and made them feel less dependent on their human therapist
Positive	Validation	10 (91)	The tone of priovi was experienced as validating, which made patients feel understood and improved their self-esteem. This was supported by priovi’s comforting daily text messages and validating case examples
Positive	Psychoeducation	10 (91)	The content was perceived as helpful, understandable, relevant, and conclusive
Positive	Specific elements	10 (91)	Some elements were mentioned as particularly helpful, including audio exercises, the regular mood check, case examples, pro-con lists, and some specific exercises
Positive	Structure	6 (55)	Positive experiences with the program’s structure related to the clear step-by-step approach, the increasing exercise difficulty, the comprehensive sequence of contents, and the easy-to-understand menu
Positive	Pleasant emotions	6 (55)	Positive emotions were, for example, induced by the nice and funny illustrations or the soothing voice of the audio speaker. Patients felt that priovi was at their side, did not abandon them, and did not force them to do anything they did not like
Negative	Technical difficulties during the pilot phase	7 (64)	These included bugs such as audio files being unavailable as text, incorrect feedback of the mood check, and temporary breakdown of the text message service
Negative	Usability problems	8 (73)	Some patients did not like specific functions, such as the duration (either too short or too long), the menu, or the voice of the audio speaker. Some patients suggested additional features, such as other items in the mood check or changes in the menu
Negative	Lack of connection with priovi	7 (64)	Some patients reported problems in relating to certain aspects of priovi, such as the digital medium in general, the comics, or the case examples. This usually improved over time—at least to some degree
Negative	Aversive emotions	5 (45)	Negative emotions occurred when patients felt confused or overwhelmed by emotionally difficult topics. Bugs or limitations of the program made some patients feel angry
Negative	Rigidity	1 (9)	There was 1 patient who found priovi too rigid and not individual enough. She felt it could not respond to her current issues well

^a^Value indicate the numbers and percentage of patients referring to the respective category.

**Table 4:** Functions and effects of priovi as reported by 6 therapists.

Function or effect	Category	n (%)^a^	Therapist experience
Helpful functions	Working materials	6 (100)	priovi was used for homework assignments and deepening the understanding of psychoeducational content
Helpful functions	Monitoring	3 (50)	The “cockpit” function was used to monitor the patients’ progress by half of the therapists
Helpful functions	Therapist representation	2 (33)	Patients could use priovi as an alternative to personal therapy in between sessions or when the therapist was on vacation
Effects of priovi	Positive effects on the therapist	6 (100)	Improved knowledge about ST, feeling supported with regard to psychoeducation, emotional relief due to priovi being there for their patients
Effects of priovi	Negative effects on the therapist	2 (33)	Obligation to motivate the patient for priovi and time burden to get familiar with priovi themselves
Effects of priovi	Positive effects on the therapy process	6 (100)	Better understanding of ST by the patient, more time for individual issues and experiential exercises, a better overall structure of therapy, improved patient responsibility
Effects of priovi	Negative effects on the therapy process	2 (33)	Discussing priovi needed therapy time
Effects of priovi	Positive effects on the patient	6 (100)	Patients learned more, learning occurred more quickly, and patients were more autonomous

^a^Value indicate the numbers and percentage of therapists referring to the respective category.

## Discussion

In this uncontrolled pilot study, we tested the feasibility and acceptability of priovi as an add-on to individual ST in 13 patients with BPD. To our knowledge, this is the first study to explore a Web-based ST tool. BPD symptoms showed improvements equaling Cohen *d*=1.0 on both BPD measures used in the study. This is rather similar to the 1-year outcome found in another study conducted in the same outpatient clinic, piloting a combination of individual and group ST [28]. In this study, mean BPDSI at baseline was 35.7 (SD 9.3), and after 1 year, it was 24.2 (SD 10.2), showing a reduction of Cohen *d*=1.2. Given these results, one could cautiously hypothesize that priovi might replace group sessions in a combined treatment format. An RCT comparing these 2 programs would be of great importance.

Most qualitative comments indicate that patients experience priovi as helpful, informative, available, and caring. Only a few adverse reactions to priovi were reported, and none of them seemed particularly severe. The majority of negative comments related to technical problems and bugs, which were immediately fixed. Therapist feedback was overall positive as well. priovi seems to be feasible, acceptable, and potentially helpful for people with BPD.

Patients used priovi intensively, but usage time was not related to clinical improvement. This is consistent with previous studies, which have suggested that usage time tends to correlate poorly with the outcome, possibly because dose-response relationships are not linear and other usage indicators, such as the number of tasks completed, might better reflect patient engagement [29]. Similarly, a linear dose-response relationship is rarely seen in psychotherapy because treatment responders may discontinue relatively early (the “good enough” effect), whereas those who are relatively treatment-resistant actually remain in treatment for longer [30]. More research is needed to disentangle the complex relationships between engagement with Web-based interventions and response.

This was mainly a qualitative study, investigating both patients’ and therapists’ experience with priovi. More structured and validated quantitative tools, such as the Client Satisfaction Questionnaire [31], would have been another option. However, we chose the interview format, since we also aimed to find unexpected or individual experiences that are not covered by the aforementioned instruments. Notably, we did not employ an elaborate qualitative methodology, such as interpretative phenomenological analysis [32], because our aim was not to understand specific experiences in great detail but rather to get a broad overview of patients’ experiences. The ultimate goal of the interviews was to improve priovi based on patient feedback and to test the acceptability and safety of priovi.

This study had several limitations. The number of subjects was small, we did not study a control group, and we only investigated priovi in combination with individual ST. Thus, we cannot make any conclusion regarding the actual efficacy of priovi on its own. Improvement in BPD symptoms might have been caused by anything, including improvement in BPD symptoms over time as has been seen in long-term studies on the course of BPD [33]. The actual efficacy of priovi needs to be demonstrated in a larger randomized controlled study.

Qualitative interviews may have elicited socially accepted response behavior since interviewers were research assistants of the study and probably positively biased toward priovi. All but 2 subjects were women, and results cannot be generalized to male patients. In general, the outpatient center in Lübeck treats severely and chronically ill patients. Thus, results cannot be generalized to patients in, for example, private practices who are often less severely ill.

## Abbreviations

BPD: borderline personality disorder
BPDSI: Borderline Personality Disorder Severity Index
DBT: dialectical behavior therapy
eHealth: electronic health
MBT: mentalization-based therapy
PD: personality disorder
RCT: randomized controlled trial
ST: schema therapy
